# An Australasian perspective on the curative treatment of patients with pancreatic cancer, supportive care, and future directions for management

**DOI:** 10.3332/ecancer.2016.700

**Published:** 2016-12-07

**Authors:** Wendy Muircroft

**Affiliations:** Southern Adelaide Palliative Service, Adelaide 5041, Australia

**Keywords:** pancreatic cancer, palliative care, cancer cachexia, pancreatic exocrine insufficiency

## Abstract

The management of patients with pancreatic cancer requires an individualised approach and the support of a multidisciplinary team to accurately stage patients and determine their suitability for curative treatment. Guidelines have been developed in Australasia to define the operability for patients who have been diagnosed with pancreatic cancer. This is supported by advances in pancreatic cancer genetics, which show potential for developing targeted therapies for pancreatic cancer. Both surgery and targeted therapies aim to extend the overall survival of patients. Patients who are cured of their cancer may live with permanent changes in gut anatomy and physiology leading to distressing symptoms that may not be addressed. Patients who cannot be cured of pancreatic cancer may have supportive care issues that are often complex, and a strategic approach to manage these needs for patients with pancreatic cancer is underdeveloped in Australasia. Supportive care services need to be in a position to adapt patient care as the evidence base develops.

## Introduction

Pancreatic cancer is a poor prognosis cancer, which had an overall 5-year survival between 1983 and 1987 of 3% in Australia [1]. Improvements in patient care have been made, which are reflected in estimates of the overall 5-year survival in Australia increasing to between 5.4% [2] and 7% in the period 2008–2012 [1]. It is projected that 6% of all deaths from cancer in 2016 will be attributable to pancreatic cancer [1].

There is currently no strategy for early detection [3]. The symptoms that lead patients to present with pancreatic cancer are often vague and patients commonly experience delays in diagnosis for a number of different reasons. There can be a lag time between presentation and completion of staging, and recommendations have been made for the completion of staging investigations within 4 weeks of presentation [3].

The net effect of these factors is that they can have an adverse impact upon patient survival, and it is estimated that in 80–90% of new cases, pancreatic cancer is irresectable at presentation [4, 5]. Patients who are eligible to undergo surgery with curative intent in Australia have a 5-year survival of 20–21% [1]. By comparison, the survival for patients who are not managed with surgery is not reported in the literature.

Within Australasia, patients with pancreatic cancer are reported to have unequal access to optimal care [6]. Recent guidelines from the Australasian Gastro-Intestinal Trials Group (AGITG) have been published with the intention of addressing some of the iniquities in service availability. The guidelines contain a consensus statement defining operable disease in pancreatic cancer [3]. For the majority of patients with pancreatic cancer who will never have curative surgery, a strategic approach to addressing their unique supportive care needs has not been defined to date in Australasia, and a multidisciplinary team-based integrated palliative care model is proposed in this paper.

### Consensus on the operability of pancreatic cancer at diagnosis

The recently published guidelines on the surgical management of pancreatic cancer from the Australasian Gastro-intestinal trials group (AGITG) places an emphasis on a multidisciplinary team (MDT) approach. These guidelines have been developed by clinicians who are members of joint Australian and New Zealand Royal Colleges and have been implemented in clinical practice in both countries. In these guidelines, the MDT makes decisions ‘about diagnosis and staging, resectability and developing a plan of management in patients for whom disease classification and setting a treatment plan is less clear’ [7].

The guidelines recommend that the following clinicians are members of a pancreatic cancer MDT:

- lead person/clinician responsible for the patient to ensure decisions made by the MDT are followed through
- specialists with an interest in pancreatic cancer (surgeon, radiologist, pathologist, radiation oncologist, gastroenterologist), allied health professionals, palliative care professional, GP, nurse coordinator, MDT coordinator, and an IT support person [7].

The AGITG consensus statement recommends referring patients to clinical trials for chemotherapy and having a mechanism for keeping track of patients who have been reviewed by an MDT, which should be undertaken to ensure recommendations were carried out and that a final outcome is known [7].

The AGITG guidelines encourage the surgical management for patients in specialist centres rather than ad hoc in private hospitals and regional centres, where there is less access to multidisciplinary review, and overall survival is poorer in Australia. Mortality figures, which originate from the United States, corroborate the AGITG recommendations as there is lower mortality in centres with the greatest numbers of patients undergoing surgery (3.8%), compared with centres with smaller practices (7.5–17.6%) [8]. There is currently no comparable data in publication from Australasia.

### Cancer genetics and the future development of targeted therapies

In 2016, the Australian Pancreatic Cancer Genome Initiative published results of the genetic analysis of pancreatic patients this year. The results of this study have led to the reclassification of pancreatic cancer into four different molecular subtypes [9]. Work is ongoing in New South Wales to make progress in the area of therapeutics to target the specific cancer subtypes.

### Identification of high-risk groups within Australasia

In New Zealand, a population-based study reporting on the outcomes of patients with pancreatic cancer has shown that Maori have higher rates of pancreatic cancer than other ethnic groups and have an unexpected female susceptibility for the disease, with the highest rates reported in any female population in the world [10]. Further, a New Zealand Ministry of Health report states that the rates of pancreatic cancer among Maori are 50–100% higher than those among non-Maori when adjusted for age [11]. There are no screening strategies to specifically identify this high-risk group and part of the increase in mortality is explained by limited health service access, and lower rates of cancer treatment [12].

The same New Zealand Ministry of Health report identified that there is also a evidence of a significant contribution from social deprivation to an increased risk of pancreatic cancer, with higher rates of pancreatic cancer in patients identified from the most socially deprived groups. Discrete figures are not cited in this report, but it is also thought that the incidence and mortality due to pancreatic cancer are approximately 5 times higher in the population group aged over 65 years than in the 45–65-year-old group [11]. When compared with Australian data, both deaths and the incidence of pancreatic cancer in New Zealand were lower than expected in comparison with Australian rates [13].

### Supportive care needs of patients with advanced pancreatic cancer

The average survival from diagnosis with metastatic pancreatic cancer was 92 days in one study published in 2002 [10]. In another study from New Zealand published in 2014, 25% of patients who were referred to a hospice service had died by day 25 of the study period, and 50% of patients had died by day 58 [14]. Given the relatively short survival from diagnosis with advanced pancreatic cancer, there is potential for patients to benefit from an early introduction to a palliative care service as a routine aspect of care. The short life expectancy from diagnosis is complicated by a high symptom burden, with some symptoms arising due to the relatively unique pathophysiological features of pancreatic cancer. These are detailed elsewhere [15]. The symptoms commonly experienced by patients include pain, weight loss, poor quality of life and high psychosocial needs [16]. Patients often have a need for the provision of end of life care with the resources provided by a palliative care multi-disciplinary team.

### Multifactorial causes of weight loss in pancreatic cancer

Weight loss is common in pancreatic cancer, and it has a negative effect upon overall survival in patients who undergo curative treatment with surgery [17]. Nutritional studies have indicated that body composition changes occur in pancreatic cancer. A study which measured body composition in operable pancreatic cancer noted that patients who had positive surgical margins had lower body fat and lean body mass. This study concluded that survival was predicted by margin status, vascular invasion, and fat mass [18]. In pancreatic cancer, weight loss can be multifactorial in aetiology and can be due to cancer anorexia-cachexia syndrome (CACS), side effects from treatment with surgery, chemotherapy or radiotherapy, nausea and vomiting, diabetes and pancreatic exocrine insufficiency (PEI).

Pancreatic cancer is unique in that significant, unintentional weight loss often occurs due to CACS and PEI. The interplay between the two conditions is not fully understood in pancreatic cancer, but there is evidence that the best treatments that are available – dietary advice, dietary supplements and pancreatic enzyme replacement therapy, are not universally available to patients at the optimal point in their disease trajectory [16]. Diabetes may occur in tumours involving either the body or the tail of the pancreas, and the presence of uncontrolled diabetes further complicates CACS and PEI by preventing weight gain when treatment with PERT and nutritional supplements are commenced.

The relatively common problem of permanent alteration in taste sensation in patients who undergo chemotherapy can complicate weight loss further, as it occurs in 21% of patients who undergo chemotherapy [19]. Patients commonly report having a metallic taste in their mouth, which alters the appeal of food and oral nutritional supplements, and may be a permanent and irreversible complication of treatment. The effects of this alteration in taste sensation affect patient compliance with oral nutrition supplements and dietary intake and needs to be monitored during chemotherapy and after completion of treatment.

Given that weight loss has a negative impact on survival, the care of patients may be compromised by disjointed assessment and treatment for weight loss. Guidelines on the management of PEI in pancreatic cancer have been available for years [20–22], but there is evidence that the condition is undertreated and this may be due to lack of physician awareness of the condition and absence of a strategic approach to the assessment and treatment of patients [14].

The assessment of clinical symptoms of malabsorption are an unreliable means of assessing the severity of the condition [23]. In the future, it may be possible to add information to diagnostic radiology and pathology with non-invasive biomarkers, which may elucidate whether there is stratification of survival with the severity of cachexia in pancreatic cancer.

### Multidisciplinary approach to the nutritional management of weight loss in pancreatic cancer

In the public health service in Australasia, dietetic resources are often focused on providing care to hospital inpatients. As a consequence, the waiting time to consult with a dietician may be as long as 2–3 months for patients with pancreatic cancer. In advanced pancreatic cancer, the waiting time between making a referral to a dietician and scheduling of an outpatient consultation can be longer than patient survival. As a consequence, some patients may not be reviewed by a dietician at any time during their illness. Dietetic services are often resourced to prioritise care for hospital in-patients, with limited support for outpatient consultations. Some dietetic services provide routine support to hepatobiliary, gastroenterology, oncology and other subspecialty services, but routine follow-up for patients is often not provided due to service constraints [16].

The management of cancer cachexia requires a multi-disciplinary approach where dietetics advice is one arm of treatment [24]. Other treatments include exercise advised by a physiotherapist and oral therapies to manage cachexia [25]. An approach such as this has been trialled in an Australian cancer centre, but it is not routinely offered to patients in all cancer centres [26]. The role of multidisciplinary cancer cachexia clinic consultation as an intervention in pancreatic cancer is an area for future research.

### The role of health care professionals in supporting patients – creating a platform for discussing patient and carer concerns about cachexia and PEI

Patients with pancreatic cancer have variable access to dietetic services, as outpatient reviews are dependent upon clinician referrals. The nutritional aspects of cancer care are not always emphasised in medical training; therefore, medical specialists caring for patients with pancreatic cancer have varying degrees in expertise at identifying which patients are at risk of cachexia before refractory cachexia is clinically apparent. Similarly, not all dieticians are familiar with the condition of PEI in pancreatic cancer and show varying familiarity with the assessment and management of patients.

Good communication between patients and health care professionals promotes compliance with treatment through greater understanding about cachexia, yet one qualitative study reported the finding of therapeutic nihilism from health care professionals towards patients with pancreatic cancer, and there was a tendency to avoid addressing weight loss and pancreatic exocrine insufficiency in consultations [16]. A systematic review of qualitative studies in cachexia reported that patients and carers wanted to have discussions with their health care providers about cachexia, as there was a tendency to make patients and carers feel isolated otherwise. The study reported: ‘as a minimum, carers want health care professionals to acknowledge patient weight loss so that they can feel confident that the problem is being taken seriously and it is a topic which is open for discussion’ [27].

### Quality of life in pancreatic cancer

The lack of information given by health care professionals about the management and symptoms caused by pancreatic cancer in general is known to have an adverse effect on quality of life in pancreatic cancer [16]. This study also identified that patients and carers needed to be informed about the symptoms that they can experience; otherwise, patients may not associate discomfort and distress they experience as arising from malabsorption, for instance. As a consequence, the conditions may not receive treatment, perpetuating quality-of-life issues.

Pancreatic cancer has effects on multiple areas of life, with social isolation described by patients due to loss of the ability to be spontaneous at making social arrangements. Patients described urgency of defaecation as a very distressing symptom [28, 29] and it made them reluctant to leave their house. Patients with this symptom feel the need to plan ahead and know where the toilets are, so-called ‘toilet dependence’. Diarrhoea often occurs after eating, which can cause faecal incontinence and may be complicated by foul-smelling wind and stools, causing embarrassment between patients, their spouses and others.

### Carer perspective on quality of life

There are quality-of-life issues that are specific to pancreatic cancer. For example, CACS can have an impact on aspects of social time with dining, shopping and the preparation of food that is subsequently discarded. In one systematic review of qualitative studies in cancer cachexia, frustration was a theme commonly expressed by carers. It was sometimes due to the inability of patients to eat the food that was prepared for them [27] and carers also found it frustrating when the food that they prepared exacerbated symptoms of PEI [16].

Carers were expressed frustration at their inability to ensure the continued well-being of the patient. ‘Taking charge of food and eating may help carers assuage feelings of powerlessness and may create an outlet for the love and care that caregivers want to provide’ [27]. Where patients could not eat the food prepared for them, conflict commonly arose between patients and carers.

### Psychosocial needs of patients and carers

The psychosocial needs of patients with pancreatic cancer and their carers are complex, and they can be related to the symptoms of the illness and also the ways in which they are treated by health care professionals. Feelings of abandonment by health care professionals have been described in the literature [16], and this has an adverse effect upon patient perception of their illness.

It has been known for some time that there is a high incidence of depression in pancreatic cancer, estimated at between 41–71% [30]. In one study, there was a higher incidence of depression recorded by score on the Hamilton Rating Scale for Depression-24 questionnaire in patients with pancreatic cancer than other upper gastrointestinal malignancies [31]. Patients also had poorer quality of life recorded with the EORTC QLQ-30 and EORTC QLQ-PAN-26 with more frequent reporting of fatigue, pain and appetite loss in the group with pancreatic cancer.

Given that there is a higher incidence of depression in patients with pancreatic cancer than in patients with other types of cancer, there is a need for access to psychiatric services to manage patients and ready access to other psychosocial services [32].

### Palliative care in pancreatic cancer – service delivery issues

#### Early referral to palliative care services

The average survival from diagnosis with metastatic pancreatic cancer was 92 days in one study published in 2002 [10], and patients are known to have unmet supportive care needs [33]. Supportive care in pancreatic cancer is not established in the routine care of patients and an argument can be made for the early referral of patients with advanced pancreatic cancer to a palliative care service. It is known that early referral for patients diagnosed with metastatic non-small cell lung cancer to a palliative care service has significant improvements upon overall survival, in quality of life and mood [34]. Temel *et al*. conducted a further study comparing the introduction of specialist palliative care visits to regular oncology care for patients with advanced non-small cell lung cancers and other poor prognosis cancers, including pancreatic cancer.

The findings from this study indicated that the group who had early access to specialist palliative care had improved quality of life, mood, ability to cope with their illness and had more frequent end of life care discussions compared with the control group [35]. There have been no equivalent studies dedicated to pancreatic cancer to definitively establish whether this is the case for patients with advanced pancreatic cancer, and as a consequence, there are no guidelines derived from randomised-controlled trial level of evidence on supportive care in pancreatic cancer. A Canadian study has indicated that palliative care is associated with less medically aggressive end of life care for patients with pancreatic cancer [36].

An integrated palliative care model could be implemented where palliative care services offer psychosocial support and symptom management while patients have chemotherapy and radiotherapy. Palliative care consult liaison services can consult when patients are inpatients and outpatient clinic consultations can take place while patients are at an ambulatory phase of their illness. When patients start to deteriorate physically or as active treatment is discontinued, the community palliative care service may then share care with the patient’s general practitioner and increase the level of community support to patients and carers as the end of life approaches.

The most frequently used model of care in Australasia is the interrupted palliative care model. The integrated palliative care model has the potential to make further improvements in symptoms experienced by patients. Each service is described in turn.

### The interrupted palliative care model

**Figure 1.**
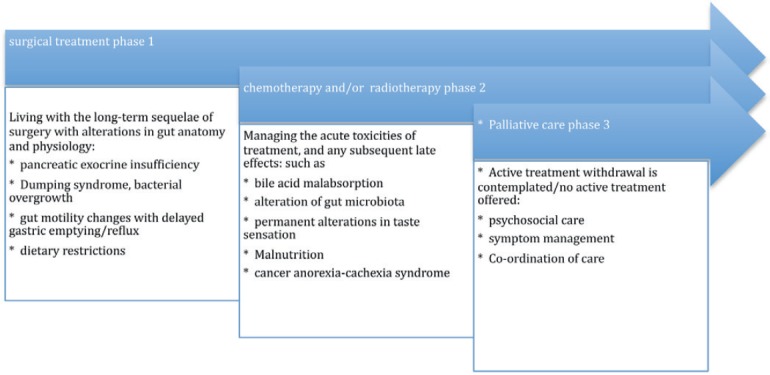
Interrupted supportive care model.

Figure 1 is a description of the palliative care model implemented in Australasia. The direction of the arrow to the right indicates the progression in time from diagnosis with pancreatic cancer to death. In this model of care, patients are referred to a palliative care service once active treatment (chemotherapy, radiotherapy) is discontinued, or plans are afoot to withdraw treatment.

Approximately, 15% of patients will be eligible to have surgery, and chronologically for this group, it will be the first treatment a patient undergoes (unless they are offered neoadjuvant chemoirradiation to downstage a tumour for operability). Curative surgery can have complications experienced by long-term survivors. The remainder of patients will have either have chemotherapy, radiotherapy or best supportive care. After undergoing multiple treatments, patients may experience symptoms due to the cumulative effects of a succession of gastrointestinal insults arising from the acute and chronic complications of treatment, such as

- chemotherapy induced diarrhoea, colitis, malabsorption, and abdominal pain [37]
- radiation enteropathy causing disruption of normal gut microbiota [38] and bile acid malabsorption [39]
- bacterial overgrowth [40], dumping syndrome and PEI arising after pancreatic surgery [41].

These treatment complications may be under-recognised by the medical teams providing the long-term follow-up for patients. Gastroenterologists also may be inexperienced at treating these conditions [28]. There is scope for specialist gastroenterology services to receive referrals to investigate and treat the complications arising from oncological therapies for pancreatic cancer. As a result, there may by improvement in the quality of life of patients, but the integration of gastroenterology services with palliative care in pancreatic cancer is not routinely provided at the current time.

### The continuous or integrated supportive care model Figure 2

This is the early referral model, where patients who can potentially gain the most from this model are those who are diagnosed with advanced disease and have a high symptom burden, psychosocial needs, and a short life expectancy.

Follow-up with the integrated palliative care services will be in the outpatient clinic for ambulatory patients with pancreatic cancer by a dedicated pancreatic cancer multidisciplinary team. This would consist of a palliative care professional, gastroenterologist, allied health care professionals, dieticians and nurse or MDT co-ordinator. As the patient’s functional status declines, referral will take place to community palliative care services and hospice or inpatient palliative care units in response to the patient’s needs. In Australasia, patients and their carers have access to domiciliary visits from palliative medicine specialists and community palliative care nurses who work in conjunction with allied health care professionals, general practitioners and district nurses. This type of supportive care model has been proposed by the Victorian State Government [42].

## Discussion

There are a number of challenges for health care professionals in the care of patients with pancreatic cancer. Patients could benefit from a strategic approach to care, which maximises their access to health care resources at a time in their disease trajectory when it can give them the most benefit.

Timely access to PERT and dietician advice for the management of CACS can be helpful for symptom relief in a subgroup of patients with PEI. Additionally, the management of PEI is important in pancreatic cancer, as it has an adverse effect upon weight. When weight loss starts to be noticeable, there is often >10-15% loss in body weight.

While there is randomised controlled trial evidence from a very small study of patients that it was possible to attenuate weight loss in patients with irresectable pancreatic cancer who were treated with PERT following the insertion of a biliary stent [43], there is no large scale, statistically significant data that indicate that refractory or established cachexia can be reversed, with either return to the patient’s pre-morbid weight, or their ideal body weight.

Pancreatic cancer is known colloquially amongst gastroenterologists in Australia as a ‘gypsy cancer’. This is based on the observation that there is often no regular surveillance of patients from a single specialist group. Gastroenterologists are often involved in the investigation and diagnosis of patients, and then, they are discharged from this service. If a patient develops biliary obstruction, a referral is made to a gastroenterology team to consider insertion of a stent for biliary drainage. By comparison, the progress of patients with hepatocellular carcinoma will be often followed up in clinic, either by a hepatologist, a gastroenterologist with an interest in this area, or by a hepatobiliary subspecialty group.

One of the advantages of having an integrated palliative care model as opposed to an interrupted palliative care model is that patients with pancreatic cancer could benefit significantly from the addition of gastroenterology follow-up to an integrated palliative care model to receive advice and specialist management of the toxicities of oncological treatment [44] and the complications of liver failure [15]. Currently, this role is provided in an ad hoc way by oncology and palliative care services, and they may not be the best services to do so.

The other potential advantages of the integrated palliative care model include the following:

- earlier access to psychosocial support services for patients and carers
- symptom management for symptoms such as pain control, nausea and vomiting
- care coordination and referrals to different specialties as needed
- anticipation and preparation for end of life care.

The provision of health care services for patients with pancreatic cancer has limitations due to resource constraints in Australasia, as there are elsewhere in the world. Any proposed changes to health care service models would benefit from economic evaluation as well as research to evaluate the risks and benefits to patients and extend the knowledge base in this area.

## Conclusion

There have been recent initiatives to streamline service delivery to patients who are diagnosed with pancreatic cancer in Australia, where the emphasis has been placed on a multi-disciplinary approach to surgery and oncological management. As a part of this initiative, early referral to a palliative care multi-disciplinary team could be encouraged for symptom management and psychosocial support in this poor prognosis cancer, where there is often evidence of poor quality of life and a short time between diagnosis and death for a significant number of patients.

## Figures and Tables

**Figure 2.**
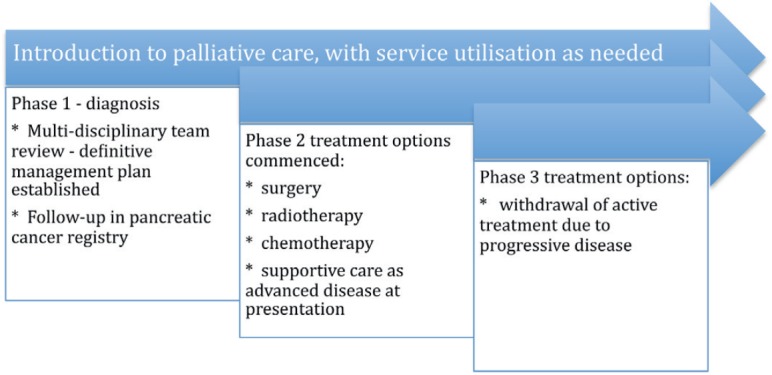
Palliative care model with continuous access.
